# FAMILY TYPOLOGY AND MENTAL HEALTH IN OLDER KOREAN AMERICANS

**DOI:** 10.1093/geroni/igae098.1566

**Published:** 2024-12-31

**Authors:** Yuri Jang, Nan Sook Park, Juyoung Park, David Chiriboga

**Affiliations:** University of Southern California, Los Angeles, California, United States; University of South Florida, Tampa, Florida, United States; USC Suzanne Dworak-Peck School of Social Work, Los Angeles, California, United States; University of South Florida, Tampa, Florida, United States

## Abstract

Challenging the notion that older adults of Asian origin are universally well protected by family support systems, we examined the heterogeneity of family types, considering both structural and qualitative aspects and addressing both positive and negative functions. The aims of the study were (1) to identify types of family relationships and (2) to examine their impact on mental health. Data came from 2,070 participants in the Study of Older Korean Americans (SOKA), a multi-state survey of Korean immigrants aged 60 and older (Mean Age = 73.3). To identify family types, latent profile analysis (LPA) was performed with marital status, living arrangement, family network, positive and negative interactions with family members, and family mistreatment. Linear regression models examined how mental distress was associated with family typology. LPA identified five family types: close-knit, intimate but distant, detached, connected but dysfunctional, and dysfunctional. Greater distress was associated with belonging to the detached, connected but dysfunctional, and dysfunctional family types in comparison to the close-knit type. Findings not only confirmed the value of a close-knit family but also demonstrated the heterogeneity of family typology. For example, the connected but dysfunctional group is uniquely characterized by those who are structurally embedded in a family system with marriage, co-residence, and contacts but had experienced negative interactions and even mistreatment within the family. This group demonstrates that obligated family ties in the absence of emotional bonding could be harmful. Our findings called renewed attention to older immigrants’ family relationships and their mental health implications.

